# Predictive biomarkers of COVID-19 impact in renal transplant patients: an exploratory proteomic and cytokine analysis

**DOI:** 10.3389/fimmu.2026.1687147

**Published:** 2026-06-12

**Authors:** Ayodele Alaiya, Maha Al-Mozaini, Zakia Shinwari, Abdulaziz Alzayed, Ibtihaj Alsharif, Rabab Allam, Razan Bakheet, Layla Alharbi, Fahad Ojab Alotaibi, Jumana Idris, Dalia A. Obeid, Abeer Alshukairi, Marcela Márquez-Méndez, Ricardo M. Cerda-Flores, Hamad A. Almojalli

**Affiliations:** 1 Biomarker Innovation for Clinical Applications, Research Laboratories Department, Riyadh, Saudi Arabia; 2 Research Laboratories Department, Riyadh, Saudi Arabia; 3 Gulf Center for Disease Prevention and Control, Riyadh, Saudi Arabia; 4 Translational Genomics Department, Centre for Genomic Medicine, King Faisal Specialist Hospital and Research Center, (KFSH&RC), Riyadh, Saudi Arabia; 5 Oncology Center, King Abdallah Center for Oncology and Liver Diseases, King Faisal Specialist Hospital and Research Center, (KFSH&RC), Riyadh, Saudi Arabia; 6 Department of Life Sciences, College of Science and General Studies, Alfaisal University, Riyadh, Saudi Arabia; 7 Transplant Research and Innovation Department, Organ Transplant Centre of Excellence, King Faisal Specialist Hospital and Research Center, (KFSH&RC), Riyadh, Saudi Arabia; 8 Department of Medicine, King Faisal Specialist Hospital and Research Center, Jeddah, Saudi Arabia; 9 Universidad Autónoma de Nuevo León, Hospital Universitario ‘Dr. José Eleuterio González’, Servicio de Oncología, Facultad de Medicina, Monterrey, Nuevo Leon, Mexico; 10 Centro de Investigación y Desarrollo en Ciencias de la Salud, Universidad Autónoma de Nuevo León, Monterrey Nuevo Leon, Mexico; 11 Kidney and Pancreas Health Center, Organ Transplant Center of Excellence, King Faisal Specialist Hospital and Research Center, (KFSH&RC), Riyadh, Saudi Arabia

**Keywords:** biomarkers, COVID-19, cytokines, immunosuppression, proteomics, renal transplant

## Abstract

**Introduction:**

Renal transplant patients (RTPs) receiving immunomodulatory therapy are at increased risk of severe complications from COVID-19 and other infections. This study aimed to identify immune and proteomic biomarkers associated with COVID-19 in RTPs to improve disease characterization and support future diagnostic and therapeutic strategies.

**Methods:**

Peripheral blood samples from RTPs with COVID-19 infection, uninfected RTPs, and healthy controls were analyzed using cytokine gene expression profiling and label-free global quantitative proteomics. Differential cytokine expression and proteomic alterations were evaluated across study groups and according to disease severity and recovery status.

**Results:**

Cytokine levels differed significantly between healthy controls and COVID-19-affected RTPs (p = 0.04), whereas no significant difference was observed between healthy controls and uninfected RTPs (p = 0.92). Within the RTP-COVID group, cytokine expression varied according to disease severity (p = 0.04) but not between acute and recovery phases (p = 0.39). Proteomic profiling identified eighteen altered protein targets. Eight proteins (SERPINF2, SAA1, HP, PLG, SERPINA3, CFHR1, HRG, and C4A) were associated with RTP-COVID and may represent candidate biomarkers of COVID-19 risk in RTPs. Ten proteins (PLXNA2, CP, A2M, KNG1, CLU, SERPINA5, APOA4, ITIH2, ITIH1, and VTN) were uniquely differentially expressed between RTPs and healthy controls but not between RTP-COVID patients and controls.

**Discussion:**

These findings provide preliminary insights into immune and proteomic dysregulation associated with COVID-19 in RTPs and identify potential biomarker candidates for disease risk and severity assessment. However, the small sample size and inclusion of only surviving patients limit the generalizability of the findings. Validation in larger and more diverse cohorts is warranted.

## Graphical Abstract

**Figure:**
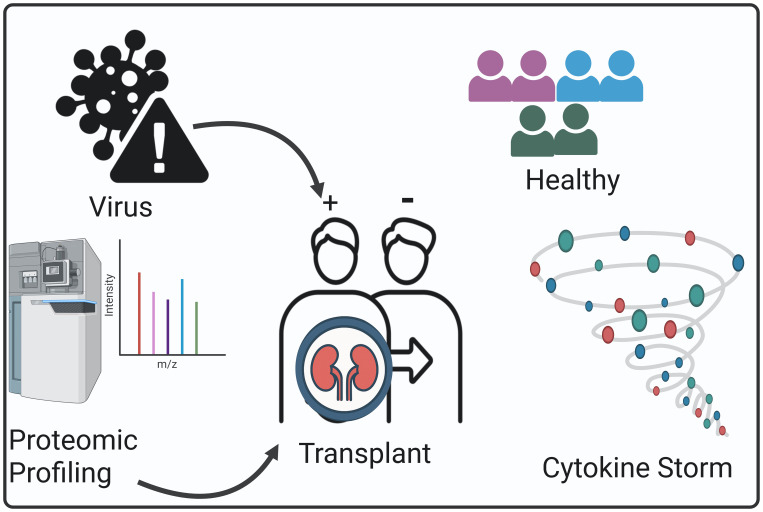

## 1 Introduction

Severe acute respiratory syndrome coronavirus 2 (SARS-CoV-2), the causative agent of COVID-19, emerged in late 2019 and rapidly evolved into a global pandemic associated with significant morbidity and mortality. Although the clinical spectrum of COVID-19 ranges from asymptomatic infection to severe respiratory failure, the leading cause of death is acute respiratory distress syndrome (ARDS) (1, 2). ARDS develops in a substantial proportion of hospitalized and critically ill patients and is strongly associated with dysregulated host inflammatory responses, particularly elevation of pro-inflammatory cytokines commonly referred to as a “cytokine storm” (1–7). This hyperinflammatory state contributes directly to alveolar damage, vascular leakage, multiorgan dysfunction, and poor clinical outcomes (1, 3–5).

SARS-CoV-2 infection initiates diverse innate and adaptive immune responses involving activation of T cells, CD4+ and CD8+ lymphocytes, and the release of pro-inflammatory cytokines and chemokines including IL-1β, IL-6, IFN-γ, MCP-1, and IP-10 (5, 6, 8, 9). Under normal circumstances, these mechanisms function to control viral replication; however, in severe disease, excessive cytokine production promotes recruitment of macrophages and granulocytes, increased neutrophil-to-lymphocyte ratios, and extensive tissue injury (8, 9). Patients with severe COVID-19 consistently demonstrate higher circulating levels of cytokines than those with moderate disease, and elevated inflammatory markers correlate with increased risk of ICU admission, need for mechanical ventilation, and mortality (3–7, 9, 10). Numerous studies have shown that critically ill patients exhibit markedly elevated IL-2, IL-6, IL-7, IL-8, IL-10, macrophage inflammatory proteins, IP-10, MCP-1, and TNF-α, along with reduced numbers of helper, cytotoxic, and regulatory T cells, reflecting profound immune dysregulation (11–14).

Solid organ transplant recipients (SOTRs), including renal transplant patients (RTPs), have been considered a particularly vulnerable population during the COVID-19 pandemic due to chronic immunosuppression, comorbidities, and impaired antiviral responses (15–17). Early clinical reports and cohort studies indicated higher rates of hospitalization, severe disease, and mortality among transplant recipients compared with immunocompetent individuals (16–23). In RTPs, COVID-19 frequently results in acute kidney injury, and a subset of patients experience graft dysfunction or loss during or after infection (16, 17, 22). Prolonged viral shedding and elevated viral loads have been described in this population, reflecting reduced capacity to clear infection effectively under immunosuppression (15). Nevertheless, some studies have proposed that baseline immunosuppressive therapy may attenuate hyperinflammatory responses and potentially mitigate cytokine-storm–mediated lung injury, illustrating the complexity of immune regulation in transplant recipients (1, 15, 18, 20).

Given the dual challenges of immunosuppression and SARS-CoV-2–induced immune activation, there is increasing interest in understanding the molecular and immunological mechanisms that contribute to disease progression in RTPs. Cytokine profiling, along with plasma proteomics, offers powerful tools to characterize immune dysregulation, identify pathways associated with severe outcomes, and elucidate biomarkers relevant to early risk stratification (24–29). These approaches may provide novel insights into the distinct immunopathology of COVID-19 in transplant populations and inform strategies to improve clinical management and graft preservation.

## 2 Results

### 2.1 Clinical characteristics

The study included six healthy individuals (median age 42), eight uninfected RTPs (median age 42), and nine RTP-COVID patients (median age 45) (**Table 1**).

**Table 1:** Clinical and demographic characteristics of subjects.

Variables	Total	Healthy controls	RTP	RTP (+) COVID-19
**Subjects n (%)**	23	6 (26)	8 (34)	9(39)
**Median age (years)**	42	42	21	45
Gender n (%)				
Female	12(52)	4 (33)	5 (41)	3(25)
Male 00	11(47)	2 (18)	3(27)	6(54)
**BMI (kg/m²), mean ± SD**		ND	23.2 ± 6.4	28.0 ± 6.9
Underlying disease				
Hypertension		*0*	*2*	
Diabetes mellitus		*0*	*2*	*1*
Others*		*0*	*4*	
COVID-19 stages				
Recovery		0	0	2
Acute		0	0	7
**Disease/Severity/ICU Admission**		0	0	6
Severe		0	0	5
Moderate		0	0	4
Outcome				
Deceased		0	0	0
Alive		6	8	9

*Other conditions as; heart, lung, and renal disease and malignancy.

The study did not measure cytokine protein levels (e.g., ELISA), limiting our ability to infer bioactive cytokine dynamics. This is acknowledged as a limitation.

Time from transplant (median year: 2018; range: 5 years).

Immunosuppressive therapy for all RTPs included a double regimen of either tacrolimus (TAC), together with mycophenolate mofetil (MMF) and prednisone.

Adjunctive COVID-19-specific therapies, treatment was administered according to institutional clinical management protocols applicable during the study period. However, given the prospective observation exploratory design and the limited cohort size, these treatment variations were not formally stratified for comparative analysis.

Cause of ESRD: hypertension, diabetes, glomerulonephritis.

All included RTPs had CMV serostatus evaluated at baseline (none had active infections).

BMI data were available only for transplant cohorts and were retrospectively retrieved from clinical records.

Renal transplantation patient RTP; Renal transplant patients infected with COVID-19 at the time of sample collection, RTP COVID-19(+).

Bold text indicates subsection headings used for organizational purposes only and does not denote statistical significance.

Limitations on subgroup analysis: Due to the limited number of RTP-COVID-19 cases with moderate disease (n=4), comparisons between moderate and severe subgroups should be interpreted cautiously. These findings are exploratory and hypothesis-generating.

#### 2.1.1 Cytokine expressions

**Figure 1:**
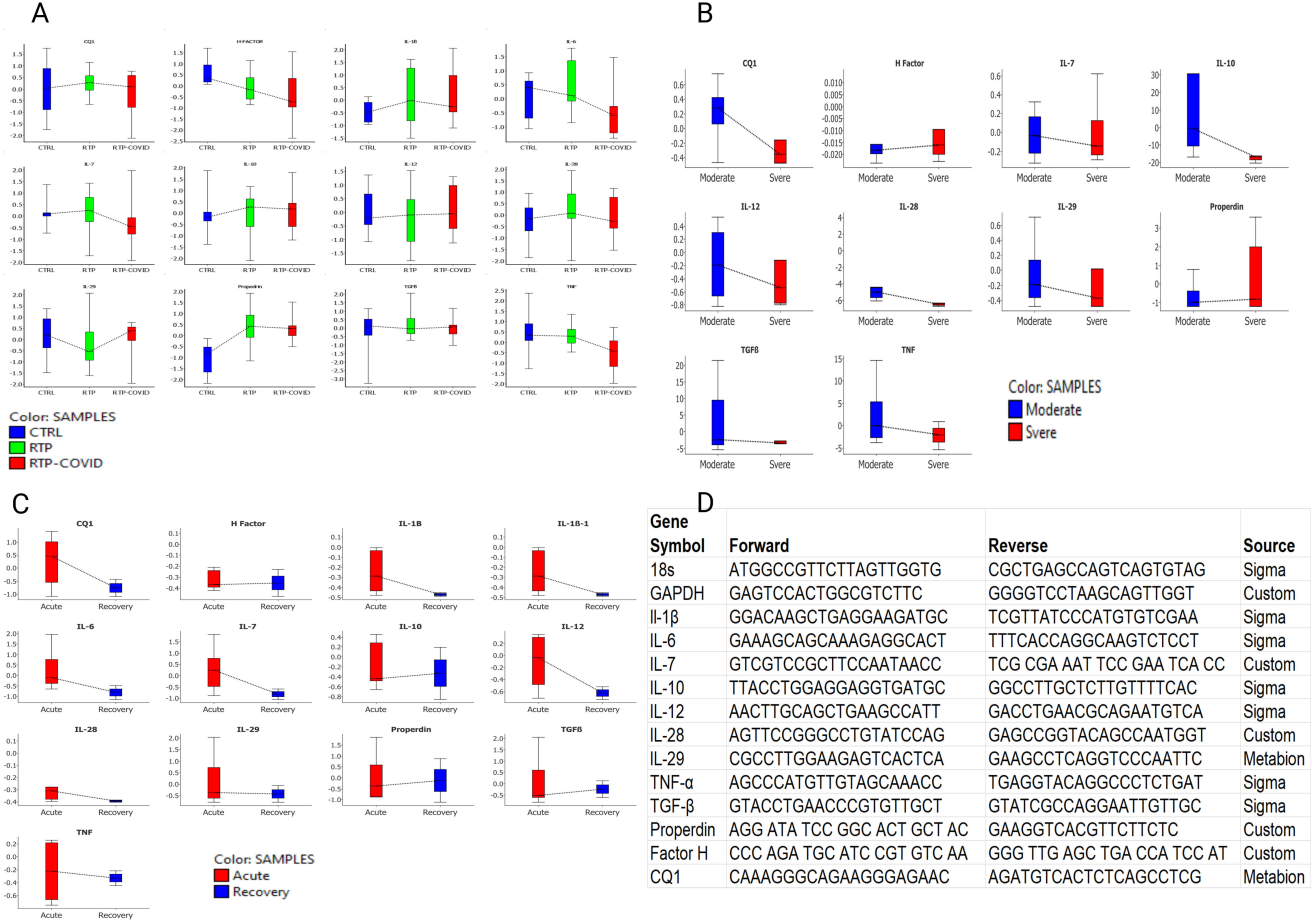
**(A)** Cytokine expression within the three populations. RNA was isolated from the PMBCS of six healthy donors, eight renal transplant patients [RTPCOVID-19 (-)), and 9 COVID-19 impacted RTPs (RTP-COVID-19 (+)). cDNA synthesis was performed using high-capacity RNA-to-cDNA commercial kits. CDNA was amplified using Sso Advanced Universal SYBR Green Supermix to detect and measure IL-1ß, IL-6, IL-7, IL-10, IL-12, IL-28, IL-29, TNF-α, TGF-ß, and Properdin expression. Assays were performed in triplicate (n=3). **(B)** Cytokine expression levels for both moderate and severe cases of COVID-19. IL-1ß, IL-6, IL-7, IL-10, IL-12, IL-28, IL-29, TNF-α, TGF-ß, and Properdin expression were assessed through gene expression analysis in the moderate vs. severe groups. **(C)** Cytokine expression in acute and recovering cases of COVID-19. IL-1ß, IL-6, IL-7, IL-10, IL-12, IL-28, IL-29, TNF-α, TGF-ß, and Properdin expression were assessed through gene expression analysis. **(D)** List of primers used in the study.

We found no significant differences in cytokine levels between healthy controls and uninfected RTPs (p=0.92). However, significant differences were observed between healthy controls and COVID-19-impacted RTPs (p=0.048) and between uninfected RTPs and the RTP-COVID cohort (p=0.039, p=0.067). The RTP cohort had higher H Factor, Properdin, and CQ1 and lower levels of IL-7 and IL-12 than healthy controls. Meanwhile, the RTP-COVID cohort had higher levels of IL-1β, IL-10, TGF-β, and H factor and lower levels of Il-7, IL-12, and IL-29, as well as CQ1 compared with healthy control subjects (**Figure 1A**).

#### 2.1.2 Moderate vs. severe RTP-COVID-19

Pairwise comparisons between disease severity and cytokine expression showed significant differences (p=0.047) among RTP-COVID-19 cohort. Of note, IL-28 was highly up-regulated within the severe group, showing elevated expression of Factor H, IL-29, Properdin, and TGF-β. Within the severe group, the levels of IL-12 and IL-1β were down-regulated compared to the moderate group (**Figure 1B**).

#### 2.1.3 Acute vs. recovering cases of COVID-19

The RTP-COVID-19 population was further analyzed for differences in cytokine expression between acute and recovering patients. Pairwise comparisons showed no significant differences in general cytokine expression for either stage (p=0.39). IL-28 was upregulated in acute samples and remained high in recovering patients. TGF-β was also most notably upregulated within the acute group compared with recovering patients. Il-29 was upregulated within the acute group but was undetectable within the recovering group (**Figure 1C**). While cytokine differences between acute and recovering RTP-COVID patients were not statistically significant, we observed trends in expression (e.g., elevated IL-28 in both phases). This may reflect persistent immune activation despite clinical recovery.

### 2.2 Proteomic profiling

**Figure 2:**
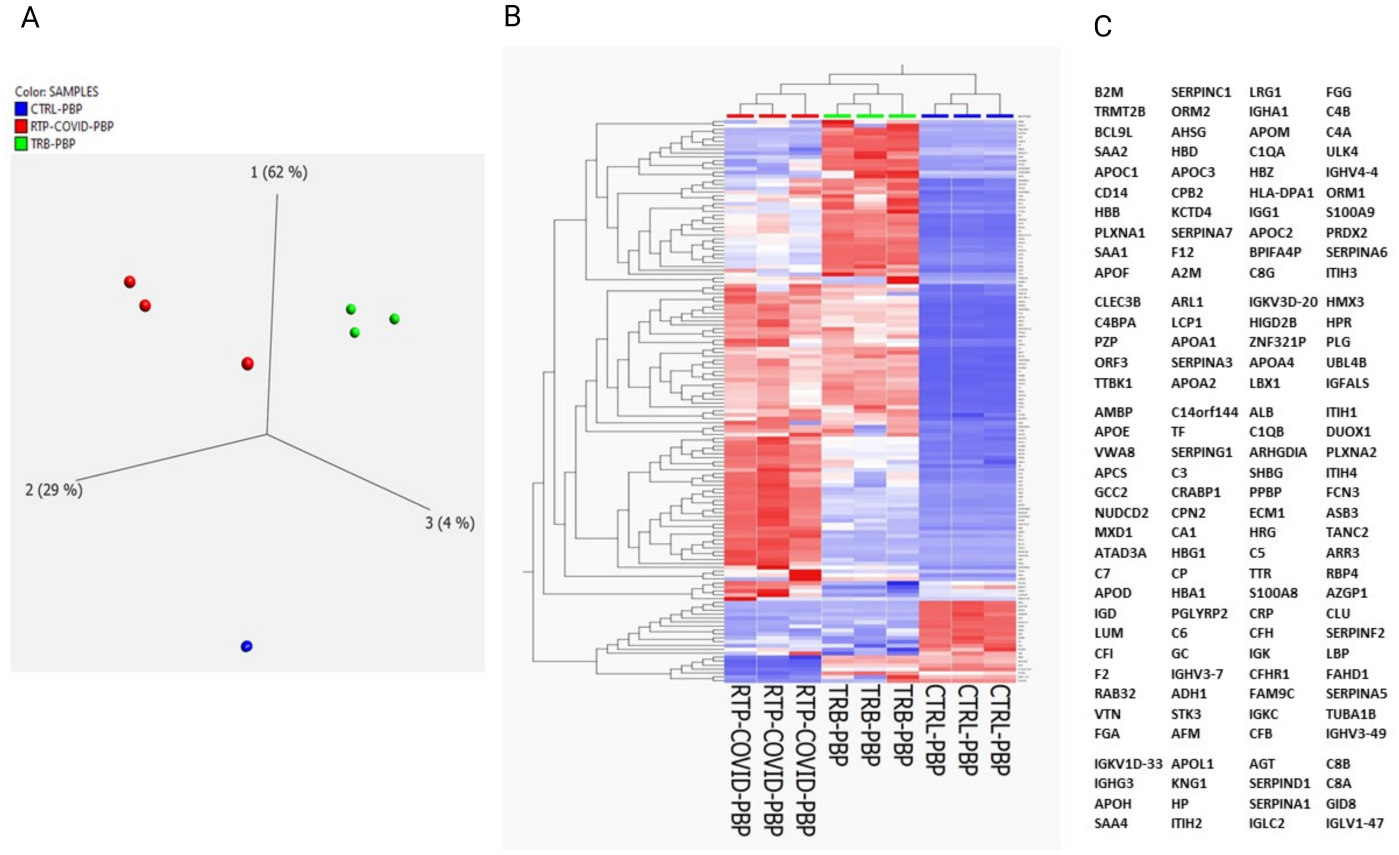
Global proteomic profiling of renal transplant patients (RTPs) and RTPs with COVID-19. **(A)** Principal component analysis (PCA) of 144 differentially expressed proteins (DEPs) across healthy controls (n = 6), uninfected RTPs (n = 8), and RTP-COVID-19 patients (n = 9). **(B)** Hierarchical clustering heat map of the same 144 DEPs, Z-score normalized across samples, illustrating relative protein abundance patterns among the three cohorts. **(C)** List of the 144 DEPs identified between RTP, RTP-COVID-19, and healthy control subjects (fold change ≥ 2; FDR-adjusted p ≤ 0.05); the complete list is provided due to limited readability within the heat map display. Heat map visualization and PCA were generated using Qlucore Omics Explorer (v3.10) (https://qlucore.com/qlucore-omics-explorer).

Only proteins meeting the predefined filtering and differential expression criteria were included in downstream comparative analyses. Across all comparisons, this yielded 162 non-redundant proteins, with 26 proteins consistently shared across all four analytical comparisons, as detailed below. Over 300 proteins were identified from all sample groups (Healthy Control, RTP &RTP-COVID-19), of which 144 were differentially expressed between the three sample cohorts (≥ 2-fold change, and ANOVA, *p* < 0·05). DEPs were subjected to unsupervised principal component analysis and hierarchical cluster analysis, resulting in three distinct sample clusters (**Figures 2A–C**).

#### 2.2.1 Sample cohorts Pair-wise proteomic analysis for discovery of disease-related DEPs

We compared the three sample cohorts to assess heterogeneity. When the pairs of RTP-COVID-19 patients and healthy control samples were compared, 118 proteins differed significantly between them. Likewise, 119 proteins were significantly dysregulated between pairs of RTP and healthy control samples.

**Figure 3:**
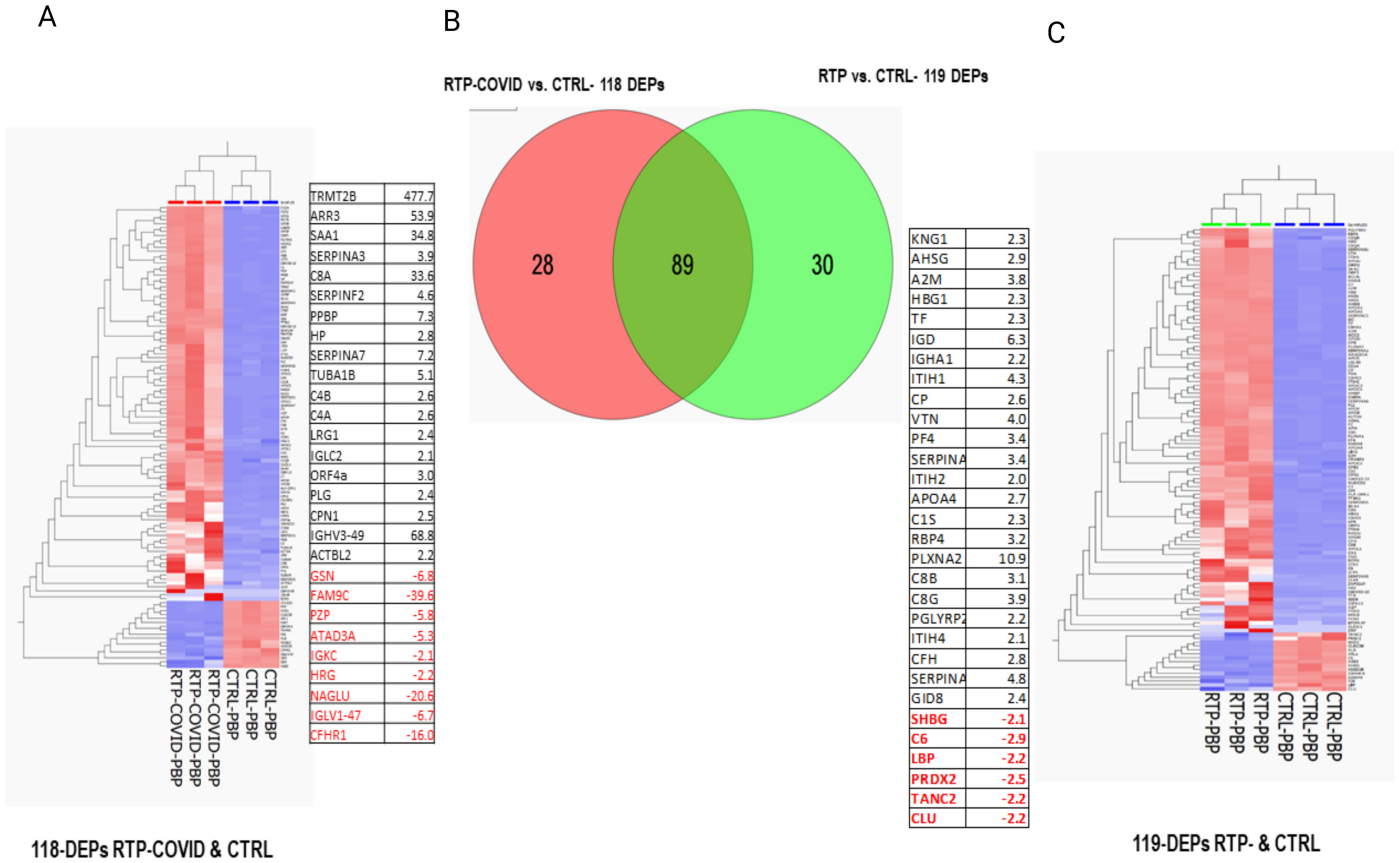
Identification of disease-specific proteomic signatures in renal transplant patients. **(A)** Hierarchical clustering heat map of 118 differentially expressed proteins (DEPs) between RTP-COVID-19 patients (n = 9) and healthy controls (n = 6), Z-score normalized across samples. The adjacent list highlights the 28 proteins uniquely differentially expressed in RTP-COVID-19 versus controls and absent in the RTP versus control comparison; proteins shown in red were up-regulated in control samples relative to RTP-COVID-19. **(B)** Venn diagram illustrating the overlap of 89 common DEPs between the RTP-COVID-19 versus control (118 proteins) and RTP versus control (119 proteins) comparisons. **(C)** Hierarchical clustering heat map of 119 DEPs between uninfected RTPs (n = 8) and healthy controls (n = 6), Z-score normalized across samples. The adjacent list highlights the 30 proteins uniquely differentially expressed in RTP versus control comparisons; proteins shown in red were up-regulated in control samples relative to RTPs. Heat map visualizations were generated using Qlucore Omics Explorer (v3.10 (https://qlucore.com/qlucore-omics-explorer). Differential expression was defined by fold change ≥ 2 with FDR-adjusted p ≤ 0.05.

Eighty-nine proteins were common between datasets, with 28 unique to RTP-COVID-19 and 30 to RTP patients (**Figure 3B**). Furthermore, only 54 proteins showed differential expression between RTPs and RTP-COVID-19 patients. The expression changes of the two RTP-COVID-19/Ctrl and RTP patients’/Ctrl cohorts’ datasets were graphically represented in distinct clusters. These dataset’s representations and expression changes are illustrated in **Figures 3A–C**).

#### 2.2.2 Identification of unique signature protein panels for each disease entity

We evaluated the overlaps between the observed 118 and 119 differentially expressed proteins between the pair-wise analysis of RTP-COVID-19 patients and healthy subjects and between RTP and healthy subjects’ samples, respectively. Only 89 proteins overlap between the 118 and 119 datasets. Interestingly, 28 proteins were uniquely expressed in COVID-19 patients, and 30 proteins were unique to RTPs only (**Figure 3B**).

#### 2.2.3 Representation of disease processes and biological interpretations of identified protein panels

To develop disease-specific biomarkers, we evaluated the two pair-wise datasets (118 and 119 DEPs). The datasets were autonomously subjected to the knowledge-based Ingenuity Pathway Analysis (IPA) to further explore their implications in protein-protein interactions, disease processes, and biological interplay in response to exposure to acute infectious agents and immune-mediated responses.

Altogether, 111 of the 118 DEPs RTP-COVID-19 patients versus healthy subjects were mapped in the IPA database. Of this, 95 and 16 of the proteins were up- and down-regulated, respectively. On the other hand, 100 and 14 proteins fulfilled the same condition from the 114 mapped of the 119 DEPs between RTP and healthy subjects. (Details of these proteins are described in **Supplementary Tables 1**, **S2**, respectively).

#### 2.2.4 Protein interactions, cellular process, and predicted network representations of the DEPs

The mapped 118 and 119 DEPs were further evaluated for their protein-protein interactions. The bulk of the 111 proteins were closely related to multiple signaling networks. The top biologically and clinically related to top disease and functions are (1) Organismal Injury and Abnormalities, Cardiac Dysfunction, Cardiovascular Disease (2) Cell-To-Cell Signaling/Interaction, Hematological System Development and Function. Others are (3) Immune Cell Trafficking, Cellular Movement, Organismal Injury and Abnormalities, and (4) Organismal Survival, Cellular Development, Cellular Growth and Proliferation.

A similar analysis was done for the 114 of 119 DEPs mapped between TRP and control samples. The top-ranked diseases and functions implicated are (i)-Organismal Injury and Abnormalities, Cardiac Dysfunction, and cardiovascular disease (ii) Endocrine System Disorders, Gastrointestinal Disease, and Immunological Disease; others are (iii) Immunological Disease, Inflammatory Disease, Organismal Injury and Abnormalities and (iv) Hematological System Development and Function, Tissue Morphology, and Cellular Movement.

The top direct disease and functional responses implicated in 111 of 118 datasets of DEP between RTP-COVID impacted and healthy control subjects are cell death, neutrophils chemotaxis, fibrinolysis, migration of vascular cells, and extent of Ca2+. Others are inflammatory response, cell movement, and molecular transport. Similar representations of the 114 of 119 DEPs between RTP and control subjects include infection by RNA virus, endothelial cell migration, lipid synthesis, and transport of molecules.

Ingenuity Pathway Analysis (IPA) mapping demonstrated that the majority of differentially expressed proteins were successfully annotated within the IPA knowledge base. Specifically, 111 of the 118 DEPs identified in the RTP-COVID-19 versus healthy control comparison and 114 of the 119 DEPs identified in the RTP versus control comparison were mapped. Canonical pathway enrichment and network analyses were performed using these mapped proteins, enabling identification of significantly enriched inflammatory, complement, metabolic, and immune-related pathways, as well as interaction networks that provide biological context to the observed proteomic changes.

Most of these proteins were in the extracellular space and cytoplasm, while a handful were in the plasma membrane and nucleus. The proteins in the two dataset panels function mainly as growth factors, transporters, enzymes, peptidases, transmembrane receptors, and transcription regulators.

**Figure 4:**
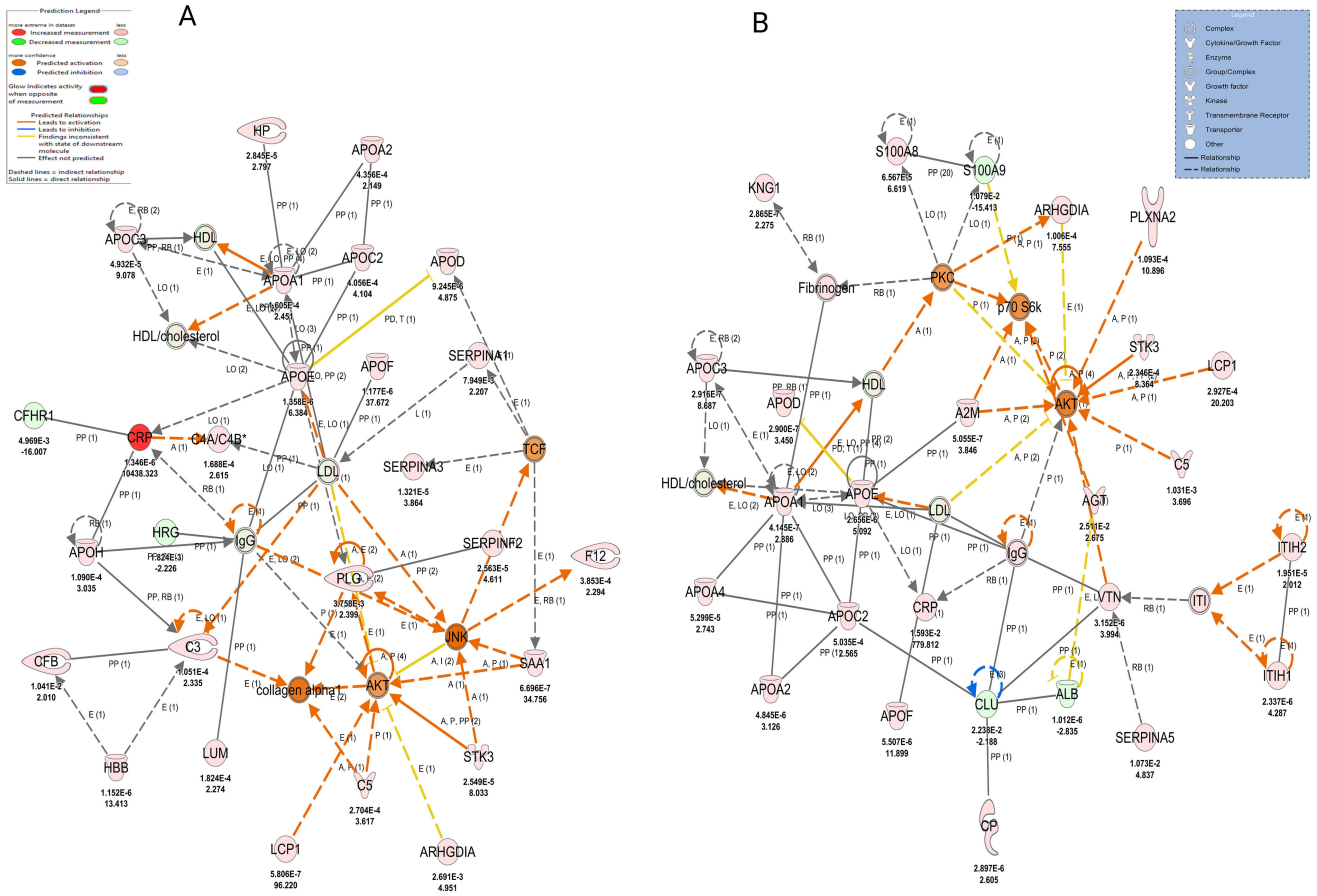
Network-level pathway analysis of differentially expressed proteins. **(A)** Knowledge-based network analysis of 111 mapped DEPs (out of 118) identified between RTP-COVID-19 patients (n = 9) and healthy controls (n = 6). The top-ranked interaction networks include inflammatory response, cell movement, molecular transport, cell death, neutrophil chemotaxis, fibrinolysis, and vascular cell migration. **(B)** Representative interaction network derived from 114 mapped DEPs (out of 119) identified between uninfected RTPs (n = 8) and healthy controls (n = 6), highlighting pathways related to RNA viral infection, endothelial cell migration, lipid metabolism, and molecular transport. Pathway and network analyses were performed using QIAGEN Ingenuity Pathway Analysis (IPA). (www.qiagen.com)).

Protein interactions and related details are shown in **Figures 4A, B** and **Supplementary Tables 1**, **S2**.

#### 2.2.5 Exploration of the established critical pathway analysis of DEPs

**Figure 5:**
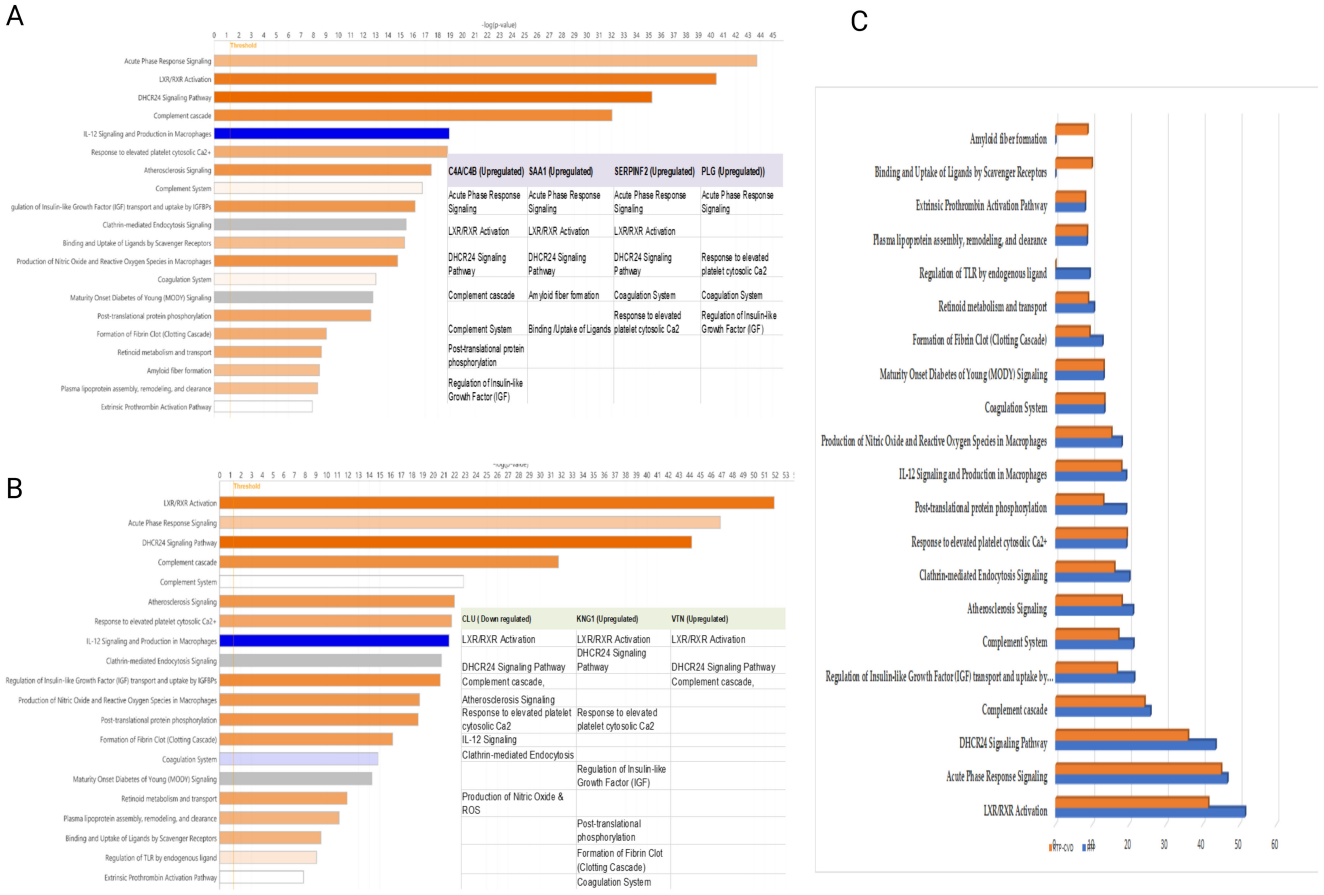
Canonical pathway enrichment analysis of proteomic signatures. **(A)** Top-ranked canonical pathways enriched among the 118 DEPs identified in RTP-COVID-19 versus healthy control comparisons, including Acute Phase Response Signaling, LXR/RXR Activation, DHCR24 Signaling, Complement Cascade, and IL-12 Signaling and Production in Macrophages. **(B)** Top-ranked canonical pathways enriched among the 119 DEPs identified in RTP versus healthy control comparisons, including LXR/RXR Activation, Acute Phase Response Signaling, DHCR24 Signaling, Complement Cascade, and Complement System. **(C)** Overlay of shared and distinct canonical pathways between the two comparisons. Canonical pathway enrichment was conducted using QIAGEN Ingenuity Pathway Analysis (IPA) based on FDR-adjusted significance.

We further explored the relationships between protein-protein interactions observed in the generated un-reviewed network, as well as the reviewed canonical pathways concerning COVID-19 and the pathophysiology of renal engraftment. Among the top-ranked three canonical pathways for the 118 datasets are Acute Phase Response Signaling, LXR/RXR Activation, DHCR24 Signaling Pathway, Complement cascade, and IL-12 Signaling and Production in Macrophages. Similarly, the topmost ranked canonical pathways for the 119 DEPs between RTP and control samples are LXR/RXR Activation, Acute Phase Response Signaling, DHCR24 Signaling Pathway, Complement cascade, and Complement System. The detailed comparison lists of the two canonical pathway representations are illustrated in **Figures 5A–C**.

#### 2.2.6 Discovery of protein panels for disease specific/related association as predictive markers for risk of renal transplant patients to COVID-19

We appraised further the 89 proteins occupying the intersection between 118 and 119 differentially expressed proteins between RTP-COVID-19 impacted versus control and 119 RTP and control subjects. Furthermore, we assessed the 28 and 30 uniquely dysregulated in each of the pairs for their representations in the two non-canonical signaling networks in **Figure 4**. The two illustrated networks represented 10 and 8 proteins among the 28 (COVID-19 markers) and 30 (Renal transplant markers) protein panels. Likewise, these sets of proteins were frequently represented in multiple top-ranked canonical pathways, as embedded in **Figure 5**.

**Figure 6:**
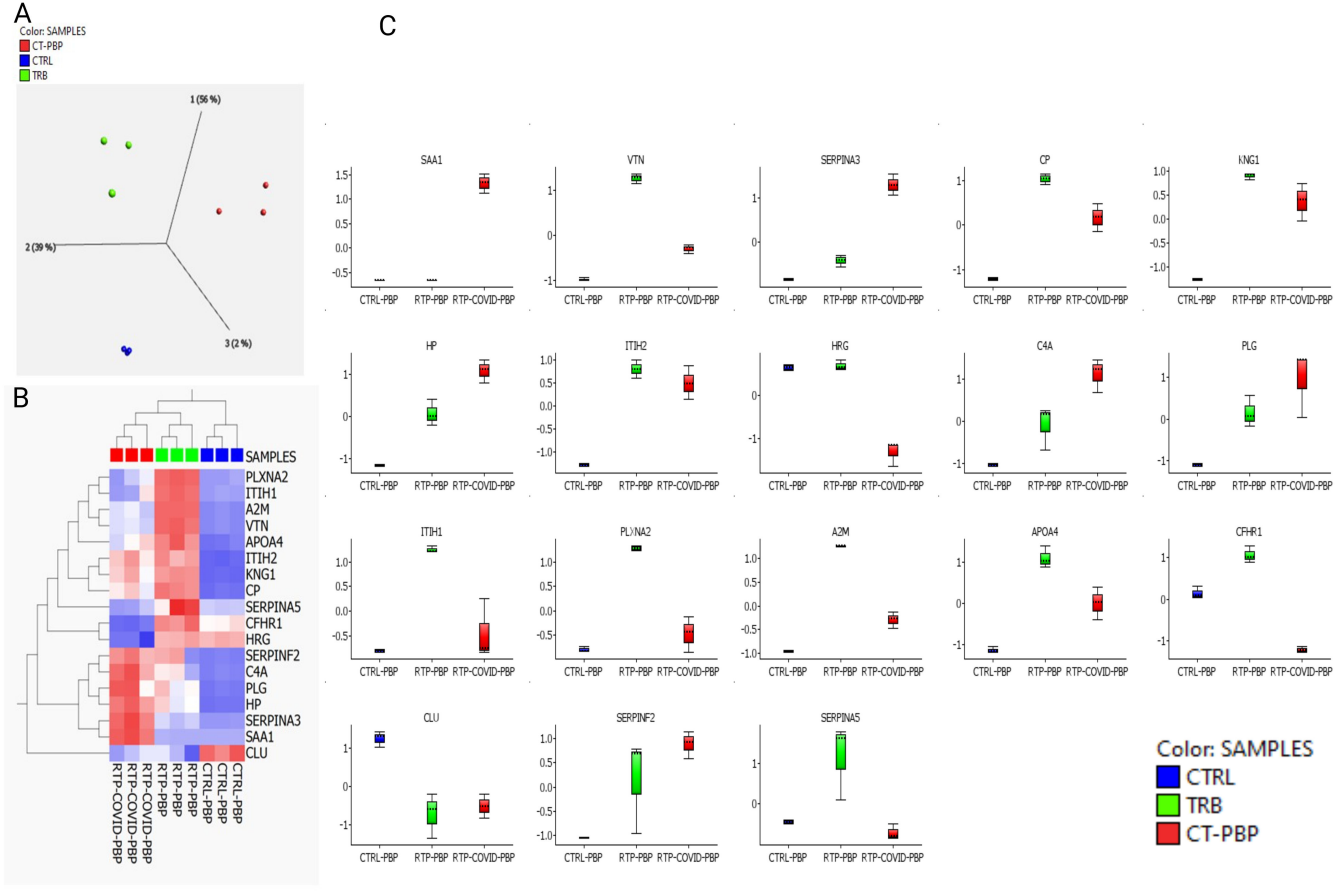
Validation and visualization of candidate predictive protein markers. **(A)** Principal component analysis (PCA) using expression profiles of the 18-protein biomarker panel across healthy controls (n = 6), uninfected RTPs (n = 8), and RTP-COVID-19 patients (n = 9). **(B)** Hierarchical clustering heat map of the same 18 proteins, Z-score normalized across samples, demonstrating cohort-specific expression patterns. **(C)** Box plots showing relative protein abundance for each of the 18 candidate markers across the three cohorts; data are presented as normalized expression values. PCA and heat map visualizations were generated using Qlucore Omics Explorer (v3.10), (https://qlucore.com/qlucore-omics-explorer).

Interestingly, following the review of the literature, most of these proteins have been previously reported in different independent studies representing COVID-19 disease and renal disease pathologies. These proteins and their expression profiles are as illustrated in **Figures 3A, C**. Additionally, the relatedness and expression changes of these proteins were represented in the PCA plot and heat map in **Figures 6A, B** respectively and their normalized quantifiable abundance expression changes of each of the 18 proteins between all three sample cohorts are represented graphically as box plots in **Figure 6C**.

### 2.3 Mechanistic model construction

To integrate these findings at a systems level, we constructed an AKT-centered mechanistic model illustrating the coordinated inflammatory, metabolic, and proteostatic pathway interactions in RTP-COVID-19 patients (**Supplementary Figure 1**). The AKT-centered mechanistic model was constructed by integrating cytokine profiling and plasma proteomic data with established signaling pathways involved in innate immune activation, complement and coagulation cascades, lipid metabolism (LXR/RXR signaling), proteostasis, and translational control. Differentially expressed proteins and cytokines identified in RTP-COVID-19 patients were mapped onto curated pathway databases and supported by published COVID-19 pathophysiology literature to generate a systems-level framework depicting inflammatory–metabolic–proteostatic crosstalk.

## 3 Discussion

Renal transplant patients (RTPs) represent a clinically vulnerable group during the COVID-19 pandemic due to their chronic immunosuppression, altered immune responses, and underlying comorbidities (18, 19). Understanding the immune alterations and proteomic disturbances associated with SARS-CoV-2 in this population remains critical for improving risk stratification and guiding therapeutic decision-making. In this study, we characterized cytokine expression and plasma proteomic signatures in RTPs with and without COVID-19, alongside healthy controls, including pre-pandemic samples to reduce confounding from immunosuppressive therapy (20). Our findings demonstrated clear differences in cytokine patterns between healthy individuals and RTPs with COVID-19, as well as between infected and uninfected RTPs. Disease severity was associated with distinct cytokine expression profiles, whereas acute and recovery-phase samples did not significantly differ, suggesting that immunosuppression or post-viral inflammation may attenuate typical temporal cytokine trajectories.

Proteomic profiling further revealed 18 differentially expressed proteins (DEPs) of interest, highlighting potential biomarkers of COVID-19 risk and severity in RTPs. Ten proteins showed unique alterations in RTPs compared with controls, whereas eight exhibited changes specifically associated with RTP-COVID-19. These protein signatures align with known pathways implicated in severe COVID-19—including acute phase response signaling, complement activation, and inflammatory cascades (24, 25, 27, 29), supporting the hypothesis that cytokine storm–related mechanisms contribute significantly to disease severity in transplant recipients.

Consistent with previous reports (21, 22), our results support a model in which severe COVID-19 arises from the interplay between direct viral cytopathic effects and dysregulated host inflammatory responses. Although immunosuppressed patients may theoretically experience milder hyperinflammatory responses due to blunted immunity (6, 12, 30), our cohort showed evidence of cytokine activation despite maintenance of baseline immunosuppressive therapy. This underscores the complex, bidirectional effects of immunosuppression: while it may impair early antiviral responses leading to higher viral loads (31), it may also temper downstream cytokine release (6), potentially moderating severe inflammatory injury. Recent clinical observations support this duality, showing that transplant recipients do not uniformly experience worse outcomes and may, in some cases, have comparable recovery to immunocompetent patients (15, 18, 32–34). The cytokine and proteomic signatures identified in our cohort provide mechanistic clues that may help clarify these apparently contradictory clinical observations. A recent proteomic investigation of peripheral blood mononuclear cells (PBMCs) from patients with COVID-19 further supports the concept of widespread host immune-metabolic reprogramming during SARS-CoV-2 infection, demonstrating perturbations in inflammatory signaling, cellular stress responses, and metabolic regulatory pathways associated with disease severity. Although that study examined intracellular immune-cell proteomic alterations rather than circulating plasma proteins, the biological themes are highly concordant with our findings, particularly regarding immune dysregulation, inflammatory amplification, and pathway-level disturbances linked to cellular stress adaptation. In the context of renal transplantation, where chronic immunosuppression further reshapes host immune responses, our plasma proteomic signatures may reflect the systemic downstream manifestation of these overlapping cellular perturbations (35).

Our analyses identified increased IL-1β, TNF-α, and IL-6 expression in RTP-COVID-19 patients, coupled with decreased IL-10, IL-12, IL-28, IL-29, IL-7, and TGF-β. These findings suggest an inflammatory imbalance characterized by heightened pro-inflammatory signaling with inadequate compensatory regulatory responses (36). Notably, IL-6—a central mediator of COVID-19–associated hyperinflammation—showed a seven-fold increase in RTP-COVID-19 patients compared with healthy controls, consistent with established correlations between IL-6 elevation and coagulopathy, organ injury, and severe disease (4). Yet IL-6 levels were paradoxically lower in RTP-COVID-19 than in uninfected RTPs, suggesting that baseline immunosuppression may modulate IL-6 induction during infection.

One of the most striking findings was decreased Complement Factor H (CFH) in RTP-COVID-19 patients. CFH tightly regulates the alternative complement pathway, and its deficiency may permit unchecked complement activation, contributing to endothelial dysfunction, microvascular thrombosis, and renal allograft injury (26, 28, 37). Reduced CFH expression in our cohort aligns with reports of complement dysregulation as a driver of severe COVID-19–related vascular pathology and suggests that RTPs may have heightened susceptibility to complement-mediated complications.

We also observed elevations in SAA1, a protein associated with renal fibrosis, inflammatory amplification, and dysfunctional HDL formation (38, 39). Marked SAA1 elevation in RTP-COVID-19 may therefore contribute to both systemic inflammation and chronic allograft injury. Additionally, increased expression of CFHR1, PLXNA2, HP, PLG, SERPINEA3, and related proteins supports prior findings implicating complement regulation, extracellular matrix remodeling, coagulation, and immune trafficking in severe COVID-19 pathogenesis (24, 25, 27, 29, 40–42). Many of the DEPs identified here have been independently associated with disease severity in COVID-19, further supporting their translational relevance.

Functional enrichment analyses revealed that DEPs in RTP-COVID-19 mapped to biological processes and disease pathways relevant to infectious disease, renal damage, cardiovascular pathology, glomerular injury, and systemic inflammation. These results highlight the multi-system impact of COVID-19 in immunosuppressed populations and underscore the need for heightened clinical vigilance. Importantly, the proteomic patterns observed in RTPs with and without COVID-19 exhibited considerable overlap, indicating that chronic immunosuppression itself drives baseline alterations in many host defense pathways. Only a subset of proteins—particularly those involved in complement regulation, acute phase response, and coagulation—distinguished COVID-19 effects from transplantation-related alterations.

A recent review of COVID-19 pathophysiology (42), highlights dysregulated innate immunity, complement activation, and hyperinflammatory cytokine signaling as central drivers of severe disease, culminating in endothelial damage, coagulopathy, microvascular thrombosis, and multi-organ injury. Our findings in renal transplant patients (RTPs) closely align with this paradigm. We observed increased pro-inflammatory cytokines (IL-1β, TNF-α, IL-6), reduced regulatory cytokines, and marked disruptions in complement regulation—most notably reduced CFH—supporting a mechanistic link between unchecked complement activation, endothelial dysfunction, and heightened thrombotic and graft-related risk in RTP-COVID-19. These abnormalities provide a molecular basis for the elevated vascular and allograft vulnerability observed in this population during SARS-CoV-2 infection.

Building on these mechanisms, we propose an expanded AKT-centered inflammatory–metabolic model (**Supplementary Figure 1**) that integrates immune activation, complement dysregulation, cytokine signaling, disrupted lipid metabolism (APOE/APOC; HDL/LDL imbalance), and proteostasis stress (PKC, p70S6K, ubiquitination) into a unified systems framework. Through its central regulatory role, AKT links these discrete pathways to drive microvascular injury, thrombosis, metabolic dysfunction, and graft damage, ultimately contributing to heightened COVID-19 severity in RTPs. Importantly, this model suggests actionable therapeutic strategies, including selective complement inhibition (e.g., C3/C5 blockade in patients with marked CFH deficiency), targeted cytokine modulation (such as IL-6 or TNF inhibition in patients with dominant hyperinflammatory phenotypes), and AKT-pathway modulation as a potential upstream strategy to simultaneously dampen inflammatory, metabolic, and proteostatic stress signaling. In addition, correction of lipid dysregulation and endothelial protection may represent adjunctive avenues for precision-based intervention in transplant-specific COVID-19 management. These integrated cytokine and proteomic perturbations are summarized in an AKT-centered mechanistic framework illustrating inflammatory–metabolic–proteostatic crosstalk driving vascular injury and graft dysfunction in RTP-COVID-19 patients (**Supplementary Figure 1**).

Together, these cytokine and proteomic findings point to a convergence of dysregulated complement activity, enhanced inflammatory signaling, and altered coagulation biology in RTP-COVID-19 patients. Such signatures may help identify transplant recipients at increased risk for severe complications and could inform personalized immunomodulatory strategies, including selective complement inhibition, cytokine blockade, or targeted adjustments to immunosuppressive regimens.

### 3.1 Study limitations

Despite the important insights provided by this study, several limitations should be acknowledged. First, the relatively small sample size (n = 23; 6 healthy controls, 8 uninfected RTPs, and 9 RTP-COVID-19) limits statistical power and generalizability. Subgroup comparisons, including analyses of moderate versus severe disease, should therefore be interpreted as exploratory and hypothesis-generating rather than confirmatory, and that future multicenter studies with larger cohorts are necessary to validate these observations. Importantly, the rarity of renal transplant recipients with well-characterized COVID-19 samples underscores the value of these preliminary findings despite the limited cohort size.

In addition, sample collection occurred at different phases of illness (7 acute and 2 recovery), which may introduce biological variability and obscure temporal immune dynamics. The identified cytokine and proteomic signatures represent associations rather than causal predictors of disease progression. Therefore, longitudinal sampling across defined disease stages would be required to fully characterize the evolution of cytokine and proteomic responses.

All RTP-COVID-19 participants in this cohort survived, which precludes identification of biomarkers associated with mortality or fulminant disease. The absence of deceased patients may restrict external validity and that future studies incorporating fatal cases will be essential to capture mortality-specific immune signatures.

Cytokine profiling was based on mRNA expression rather than protein-level quantification, and given the potential discordance between transcript and protein abundance, future studies using ELISA, multiplex platforms, or targeted proteomics are warranted.

Immunosuppressive therapy represents a potential confounding factor in immune and proteomic profiling of transplant recipients. In the present study, all renal transplant patients were maintained on broadly comparable baseline immunosuppressive regimens, consisting of tacrolimus- -based therapy in combination with mycophenolate mofetil and prednisone, and no major dosage adjustments were made during the course of COVID-19 infection. These clinical details are described in the Materials and Methods section.

However, although treatment regimens were generally similar, inter-individual variability in immunosuppressive exposure and time since transplantation may still have influenced cytokine and proteomic expression patterns. Stratification according to specific immunosuppressive agents or drug levels would have provided additional resolution but was not feasible due to the limited cohort size. Future studies with larger, well-powered cohorts should incorporate stratified or drug-specific analyses to more precisely delineate the immunomodulatory effects of individual immunosuppressive therapies on SARS-CoV-2–associated immune responses.

Furthermore, heterogeneity in time from transplantation, baseline immunosuppressive regimens, comorbid conditions, and medication adjustments may influence immune and proteomic signatures. An additional limitation is that BMI data were only available for the transplant cohorts and not for healthy controls. Consequently, BMI was not included as an adjustment variable in the exploratory proteomic analysis. Given the known influence of adiposity on inflammatory and metabolic pathways, a potential confounding effect of BMI on observed proteomic changes cannot be excluded. Although these variables were documented and considered, residual confounding cannot be excluded. Finally, the single-center study design and lack of SARS-CoV-2 variant identification may limit the broader generalizability of these findings, and plasma-based analyses may not fully reflect tissue-level processes within the allograft or respiratory tract.

## 4 Materials and methods

### 4.1 Study design and subjects

This prospective observational study analyzed plasma from RTPs with varying COVID-19 severity. To exclude any impact of immunosuppressant therapy, samples were compared with those collected from transplant patients before the COVID-19 pandemic. Healthy subjects were the control group.

#### 4.1.1 Patient selection

##### 4.1.1.1 Sample selection and size rationale

Kidney transplant recipients (KTRs) with confirmed SARS-CoV-2 infection were identified during routine clinical care, and peripheral blood samples were collected during clinically active COVID-19 infection. The RTP-COVID-19 cohort comprised kidney transplant recipients with clinically confirmed COVID-19, including predominantly severe cases requiring ICU admission.

The RTP comparator cohort consisted of SARS-CoV-2–negative kidney transplant recipients sampled during routine clinical follow-up. Both RTP-COVID-19 and RTP comparator samples were collected within 6–12 months post-transplantation to improve comparability with respect to transplant timing. Where feasible, cohort selection also considered demographic characteristics including age and sex.

Healthy controls were SARS-CoV-2–negative individuals without renal disease. Participants were included if appropriate biospecimens were available and informed consent for research use had been obtained. The final cohort reflected all eligible samples meeting these criteria during the study period. Given the exploratory pilot nature of the study, no formal sample size calculation was performed.

Disease severity was classified based on WHO guidelines (**Supplementary Table 3**: “moderate” defined as pneumonia with no signs of severe disease; “severe” defined as oxygen saturation < 90% or signs of respiratory distress. as previously described (24). The disease stage refers to the patient’s status during sample collection. Acute refers to symptom presence during sample collection; recovery refers to asymptomatic status with virus clearance. Immunosuppressive therapy for all RTPs included a double regimen of either tacrolimus (TAC) or tocilizumab, together with mycophenolate mofetil (MMF) and prednisone. During COVID-19, all patients continued their immunosuppressant regimens without dosage adjustments. All included RTPs had CMV serostatus evaluated at baseline (none had active infections). All patients provided informed consent. The institutional review board approved the protocol (KFSHRC RAC # 2180025). The criteria for patient selection were COVID-19-impacted patients who underwent kidney transplants at the KFSHRC.

### 4.2 Blood sample collection and preparation

Blood samples were collected in EDTA tubes and processed into leukocytes and plasma. Samples were stored in a Biobank before processing.

### 4.3 RTP COVID-19 patients

Individuals positive for COVID-19 were confirmed by both clinical observation (symptoms) and categorized according to the Guidelines for Diagnosis and Management of Coronavirus Disease 2019 (24). Positive PCR swab results were confirmed by a diagnostic laboratory at our institution. Clinical and demographic data were recorded, including the date of ICU admission and patient outcomes (**Table 1**). All the laboratory test results done are presented in **Table 2**.

**Table 2:** Laboratory investigations of subjects.

Laboratory investigations Parameters	COVID-19 impacted renal transplant patients (RTP COVID +)									Renal transplant patients (RTP)							
	RTP CVD1	RTP CVD2	RTP CVD3	RTP CVD4	RTP CVD5	RTP CVD6	RTP CVD7	RTP CVD8	RTP CVD9	RTB1	RTB2	RTB3	RTB4	RTB5	RTB6	RTB7	RTB8
Gender	F	M	M	M	M	F	M	F	M	M	F	F	M	F	M	F	F
Age	38	79	34	29	57	58	72	36	31	20	18	18	14	21	18	19	16
RBC (10^12/L)	3.86	4.68	4.94	4.91	3.49	5.16	5.73	5.82	3.76	4.92	5.11	3.88	4.83	4.33	4.95	3.52	4.37
WBC (10^9/L)	11.27	2.19	8.76	6.22	1.93	12.03	4.59	7.37	1.33	9.64	6.88	5.52	7.08	7.65	5.98	9.74	6.12
Hematocrit (L/L)	0.321	0.421	0.414	0.432	0.261	0.429	0.469	0.437	0.327	0.423	0.43	0.306	0.416	0.367	0.417	0.254	0.372
Hemoglobin (g/L)	102	142	137	136	80	149	151	140	117	142	148	99	123	117	138	80	115
Monocyte Abs (10^9/L)	0.99	0.28	0.99	0.4	0.08	0.68	0.61	0.36	0.81	0.58	0.3	0.06	0.94	0.92	0.57	1.04	0.5
Lymphocyte Abs (10^9/L)	0.9	0.21	2.79	1.18	0.16	0.86	1.91	2.71	0.28	3.86	4.03	3.38	1.92	3.6	2.55	1.65	3.19
Neutrophil abs (10^9/L)	9.73	1.7	4.08	4.62	1.43	10.46	1.95	4.2	0.38	4.72	2.91	2.06	4.03	2.93	2.68	6.52	2.08
Platelet (10^9/L)	122	107	225	251	395	325	175	195	61	402	281	375	275	318	289	481	278
Albumin (g/l)	37	34	42	46	29	38	30	42	30	45.8	39	41.1	42.8	41.6	44.1	38	41.6
LDH (U/L)	269	222	196	221	305	198	183	198	279	N/A	N/A	496	N/A	N/A	N/A	N/A	N/A
Ferritin (µg/l)	491	2087	580	234	5942	936	201.8	61.2	442	N/A	N/A	N/A	N/A	N/A	N/A	317	N/A
CRP (mg/l)	8.48	29.3	10.24	12.2	344	14.49	9.07	6.38	308.38	N/A	N/A	66.8	N/A	N/A	N/A	113.4	N/A
D-Dimer (mg/l)	0.27	0.71	0.17	0.33	0.54	0.24	0.32	N/A	9.18	N/A	N/A	N/A	N/A	N/A	N/A	N/A	N/A
Procalcitonin (ng/ml)	0.1	0.13	0.06	N/A	0.32	0.14	0.04	0.06	N/A	N/A	N/A	N/A	N/A	N/A	N/A	N/A	N/A
Alkaline Phosphate (U/L)	121	57	74	135	131	102	51	85	76	193	130	146	240	84	334	86	337
AST (U/L)	75	33	20	12	12	13	14	15	34	16.2	18.2	12.7	34.9	16	19.2	14.7	18.2
ALT (U/L)	127	12	17	7	16	15	14	14	24	12.8	13.5	8	23.9	16.7	12.5	6	14
Creatinine (µmol/L)	76	80	97	184	114	76	83	105	253	63	95	63	68	39	61	567	87

*Data presented represents individual values. RTP, Renal Transplant Patient; CVD, COVID-19; CRP, RBC, Red Blood Cell; WBC, White Blood Cell; LDH, Lactate Dehydrogenase; CRP, C-Reactive Protein; AST, Aspartate Aminotransferase; ALT, Alanine Transaminase; NA, Not Available.

### 4.4 RNA extraction and gene expression analysis

RNA was extracted from white blood cells (PMBCs) of six healthy controls, eight RTPs, and nine RTP-COVID patients using TRIzol and quantified with a Nanodrop 7500. cDNA synthesis was performed using high-capacity RNA-to-cDNA commercial kits (ThermoFisher). cDNA was amplified using Sso Advanced Universal SYBR Green Supermix (BioRad) to detect and measure IL-1ß, IL-6, IL-7, IL-10, IL-12, IL-28, IL-29, TNF-α, TGF-ß, and Properdin expression. Samples were probed for the housekeeping genes 18s and GAPDH. Assays were performed in triplicate using the CFX96 Real-Time System (Bio-Rad).

#### 4.4.1 Primer design

Primers were designed using the nucleotide Basic Local Alignment Search Tool (BLAST) (http://blast.ncbi.nlm.nih.gov/Blast.cgi). Select primers were designed in-house using the oligonucleotide synthesis laboratory at KFSHRC. The primer sequences are shown in (**Figure 1D**).

### 4.5 Data collection

All patients’ clinical data were extracted using assigned numbers from KFSHRC electronic medical records. Clinicians were blinded to the outcome of cytokine profiling and cytokine storm dynamics data were analyzed. Demographic characteristics were recorded, including comorbidities, symptom onset data, reported symptoms, treatment regimens, prognosis, and disease severity (**Table 1**). Comorbidities included diabetes mellitus, hypertension, heart disease, renal failure, congenital nephrotic syndrome, and receipt of immunosuppressive medications. The severity of the illness was evaluated according to the 2019 Guidelines for Diagnosis and Management of Coronavirus Disease as described previously (24) **Supplementary Table 3**.

### 4.6 Proteomics and mass spectrometry analysis

Plasma from three subject groups were pooled (healthy subjects, kidney transplant without COVID-19, and kidney transplant with COVID-19). Peptide digestion was analyzed using one-dimensional Nano Acquity liquid chromatography coupled to Synapt G2 HDMS on a Trizaic Nano-flow source (Waters, Manchester, UK). Proteomic data were generated between the sample groups as previously described (43, 44). MS data were acquired in the range of m/z 50–2000 Da for 120 min gradient acquisition using ion mobility separation experiments (HDMSE). Samples were analyzed in triplicate using the Mass Lynx platform (version. 4·1, SCN833).

### 4.7 Data analysis

The qRT–PCR data were analyzed using the Livak & Schmittgen or 2-ΔΔCT method. The Ct values were normalized to GAPDH, and fold changes were calculated. Data were analyzed using R statistical software (version 4.0.3). Pairwise t-tests were performed on the differences between the two groups. A Type II ANOVA correlation test was performed for differences between all three populations and between the moderate vs. severe and acute vs. recovery groups within the RTP-COVID-19 cohort. P values <0.05 were deemed significant.

Label-free relative quantification of the proteomics data was performed using Progenesis QI for Proteomics (Nonlinear Dynamics, Waters). This software performs normalization across all LC-MS runs by aligning retention times and applying global scaling to correct for systematic variation between runs. This ensures consistency and comparability of protein abundance measurements across the three sample groups. We have used the FDR-based approach in Progenesis QI to minimize Multiple-testing correction by integrating statistical models that estimate q-values for each protein based on Benjamini–Hochberg correction. In our analysis, we considered proteins with an adjusted p-value (q-value) ≤ 0.05 and a fold change ≥ 2 as statistically significant. This dual threshold was used to both control for false positives and emphasize biologically relevant changes. To further strengthen statistical rigor, we also evaluated the power of detection, using Progenesis’ built-in power analysis tools, and excluded proteins falling below a minimum power threshold, as recommended to mitigate issues of overfitting and inflated FDR in large datasets.

In support of data transparency, all raw MS data and the complete list of identified proteins (with associated quantification metrics) have been deposited in the PRIDE database via the ProteomeXchange Consortium (accession: [PXD063262 and 10.6019/PXD063262]).

## 5 Conclusions

Despite these limitations, this study provides valuable insight into the cytokine and proteomic alterations associated with COVID-19 in renal transplant recipients. We identified several candidate biomarkers involved in acute phase response signaling, complement regulation, coagulation, lipid metabolism, and immune modulation. These findings, which align with emerging reports of COVID-19 pathogenesis in immunocompromised populations, highlight biological pathways that may be leveraged for early risk assessment and therapeutic intervention. Larger, longitudinal, multicenter studies are needed to validate these candidate markers and further elucidate the mechanisms underlying COVID-19 severity in transplant recipients.

## Data Availability

The datasets presented in this study can be found in online repositories. The names of the repository/repositories and accession number(s) can be found in the article/**Supplementary Material**.

## Supplementary material

Supplementary Table 1 111 of the 118 DEPs between RTP-COVID-19 patients versus healthy subjects that were mapped in the IPA database.

Supplementary Table 2 114 of the 119 DEPs between RTP patients versus healthy subjects that were mapped in the IPA database.

Supplementary Table 3 Table COVID-19 Disease Severity Classification (Simplified WHO Guidance).

Supplementary Figure 1 AKT-centered mechanistic model of inflammatory–metabolic–proteostatic crosstalk in renal transplant patients with COVID-19. SARS-CoV-2 infection triggers innate immune activation, leading to complement and coagulation pathway activation, acute-phase reactant production, and endothelial stress. These upstream inflammatory signals converge on AKT as a central regulatory hub, integrating inflammatory crosstalk, metabolic adaptation, and translational control. Dysregulation of LXR/RXR signaling and lipid metabolism contributes to vascular injury, while activation of the PKC–p70S6K axis promotes proteostasis imbalance and cellular stress responses. These interconnected pathways collectively drive microvascular dysfunction, thrombosis, metabolic derangement, and graft injury, resulting in heightened COVID-19 severity in renal transplant patients. This integrative model highlights AKT, complement pathways, and translational stress signaling as actionable therapeutic targets.
