# Supplementary material for: Predictive biomarkers of COVID-19 impact in renal transplant patients: an exploratory proteomic and cytokine analysis
Source: Front Immunol. 2026 Jun 12;17:1687147. doi: 10.3389/fimmu.2026.1687147 (PMC13303355; doi:10.3389/fimmu.2026.1687147)
Supplement: Supplementary Table 3 — Table COVID-19 Disease Severity Classification (Simplified WHO Guidance). [file Table2.docx]

# Supplementary Table S3-COVID-19 Disease Severity Classification (Simplified WHO Guidance)

| **Category** | **Clinical Features** | **Key Indicators** |
| --- | --- | --- |
|  |  |  |
| Mild | - Symptoms of COVID-19 (e.g. fever, cough, fatigue, loss of taste/smell) without signs of pneumonia or hypoxia. | - No shortness of breath - Oxygen saturation (SpO₂) ≥ 95% on room air - No clinical signs of pneumonia |
|  |  |  |
| Moderate | - Clinical signs of pneumonia without signs of severe disease. | - Cough, fever, fast breathing - SpO₂ 90–94% on room air - No signs of severe respiratory distress |
|  |  |  |
| Severe | - Severe pneumonia or other critical conditions like ARDS, sepsis, or septic shock. | - SpO₂ < 90% on room air - Respiratory rate > 30/min (adults) - Signs of severe respiratory distress (e.g. use of accessory muscles, inability to complete sentences) - Cyanosis, confusion, hypotension |
