# Supplementary figures and images for: Predictive biomarkers of COVID-19 impact in renal transplant patients: an exploratory proteomic and cytokine analysis
Source: Front Immunol. 2026 Jun 12;17:1687147. doi: 10.3389/fimmu.2026.1687147 (PMC13303355; doi:10.3389/fimmu.2026.1687147)

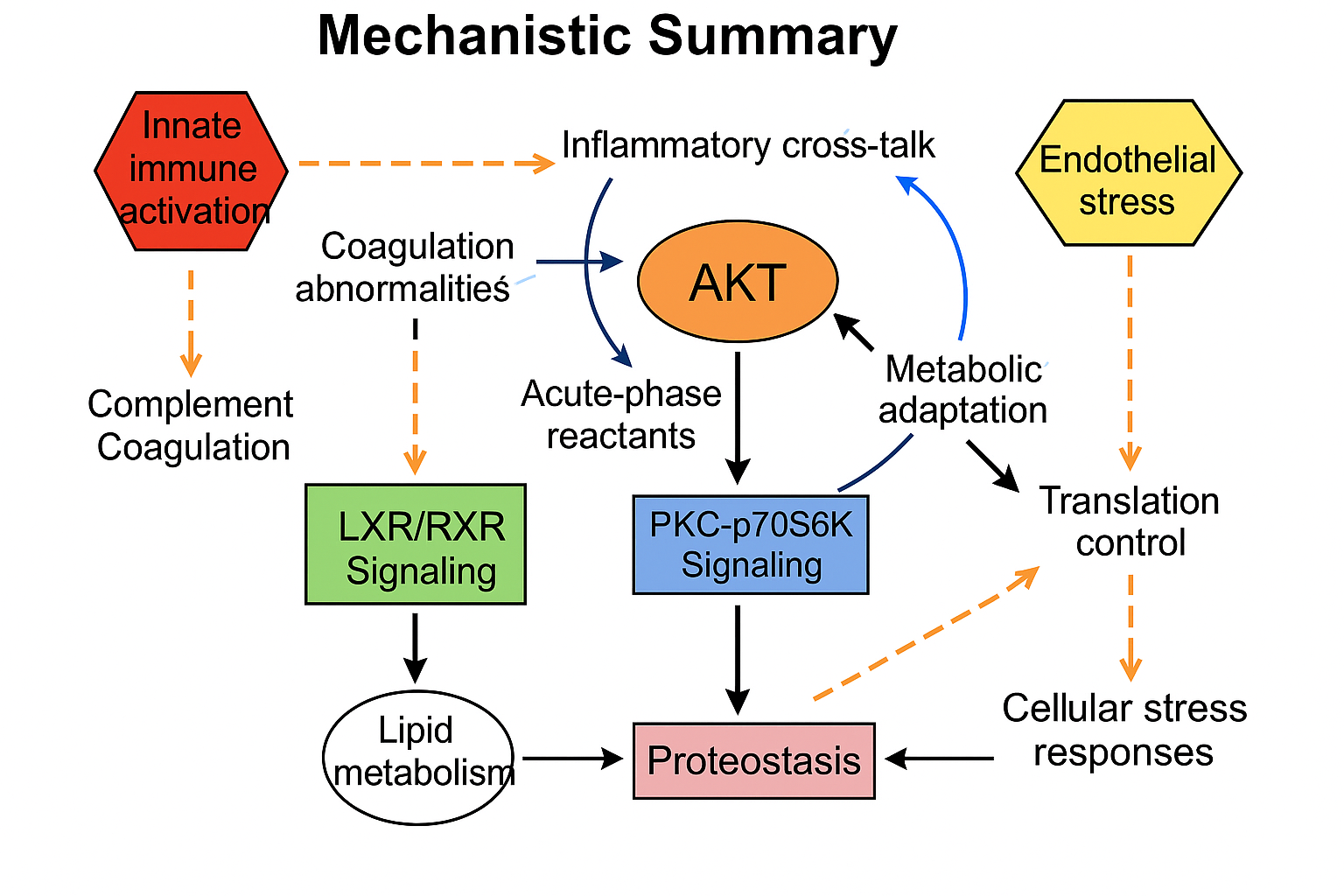

Supplement: Supplementary Figure 1 — AKT-centered mechanistic model of inflammatory–metabolic–proteostatic crosstalk in renal transplant patients with COVID-19. SARS-CoV-2 infection triggers innate immune activation, leading to complement and coagulation pathway activation, acute-phase reactant production, and endothelial stress. These upstream inflammatory signals converge on AKT as a central regulatory hub, integrating inflammatory crosstalk, metabolic adaptation, and translational control. Dysregulation of LXR/RXR signaling and lipid metabolism contributes to vascular injury, while activation of the PKC–p70S6K axis promotes proteostasis imbalance and cellular stress responses. These interconnected pathways collectively drive microvascular dysfunction, thrombosis, metabolic derangement, and graft injury, resulting in heightened COVID-19 severity in renal transplant patients. This integrative model highlights AKT, complement pathways, and translational stress signaling as actionable therapeutic targets. [file Image1.png]
